# Variability in grading of ductal carcinoma *in situ* among an international group of pathologists

**DOI:** 10.1002/cjp2.201

**Published:** 2021-02-23

**Authors:** Maartje van Seijen, Katarzyna Jóźwiak, Sarah E Pinder, Allison Hall, Savitri Krishnamurthy, Jeremy SJ Thomas, Laura C Collins, Jonathan Bijron, Joost Bart, Danielle Cohen, Wen Ng, Ihssane Bouybayoune, Hilary Stobart, Jan Hudecek, Michael Schaapveld, Alastair Thompson, Esther H Lips, Jelle Wesseling, The Grand Challenge PRECISION consortium.

**Affiliations:** ^1^ Division of Molecular Pathology The Netherlands Cancer Institute Amsterdam The Netherlands; ^2^ Institute of Biostatistics and Registry Research Brandenburg Medical School Theodor Fontane Neuruppin Germany; ^3^ Comprehensive Cancer Centre at Guy's Hospital, School of Cancer & Pharmaceutical Sciences Kings College London London UK; ^4^ Department of Cellular Pathology Guy's and St Thomas' NHS Foundation Trust London London UK; ^5^ Department of Pathology Duke University Medical Center Durham NC USA; ^6^ Department of Pathology The University of Texas MD Anderson Cancer Center Houston TX USA; ^7^ Department of Pathology Western General Hospital Edinburgh UK; ^8^ Department of Pathology Beth Israel Deaconess Medical Center and Harvard Medical School Boston MA USA; ^9^ Department of Pathology Martini Hospital Groningen The Netherlands; ^10^ Department of Pathology Isala Hospital Zwolle The Netherlands; ^11^ Department of Pathology University of Groningen, University Medical Center Groningen Groningen The Netherlands; ^12^ Department of Pathology Leiden University Medical Center Leiden The Netherlands; ^13^ Independent Cancer Patients' Voice London UK; ^14^ Department of Research IT The Netherlands Cancer Institute Amsterdam The Netherlands; ^15^ Department of Psychosocial Research and Epidemiology The Netherlands Cancer Institute Amsterdam The Netherlands; ^16^ Dan L Duncan Comprehensive Cancer Center Baylor College of Medicine Houston TX USA

**Keywords:** ductal carcinoma *in situ*, pathology, grade, interobserver variability

## Abstract

The prognostic value of cytonuclear grade in ductal carcinoma *in situ* (DCIS) is debated, partly due to high interobserver variability and the use of multiple guidelines. The aim of this study was to evaluate interobserver agreement in grading DCIS between Dutch, British, and American pathologists. Haematoxylin and eosin‐stained slides of 425 women with primary DCIS were independently reviewed by nine breast pathologists based in the Netherlands, the UK, and the USA. Chance‐corrected kappa (*κ*_ma_) for association between pathologists was calculated based on a generalised linear mixed model using the ordinal package in R. Overall *κ*_ma_ for grade of DCIS (low, intermediate, or high) was estimated to be 0.50 (95% confidence interval [CI] 0.44–0.56), indicating a moderate association between pathologists. When the model was adjusted for national guidelines, the association for grade did not change (*κ*_ma_ = 0.53; 95% CI 0.48–0.57); subgroup analysis for pathologists using the UK pathology guidelines only had significantly higher association (*κ*_ma_ = 0.58; 95% CI 0.56–0.61). To assess if concordance of grading relates to the expression of the oestrogen receptor (ER) and HER2, archived immunohistochemistry was analysed on a subgroup (*n* = 106). This showed that non‐high grade according to the majority opinion was associated with ER positivity and HER2 negativity (100 and 89% of non‐high grade cases, respectively). In conclusion, DCIS grade showed only moderate association using whole slide images scored by nine breast pathologists. As therapeutic decisions and inclusion in ongoing clinical trials are guided by DCIS grade, there is a pressing need to reduce interobserver variability in grading. ER and HER2 might be supportive to prevent the accidental and unwanted inclusion of high‐grade DCIS in such trials.

## Introduction

Ductal carcinoma *in situ* (DCIS) is a non‐obligate precursor of invasive breast cancer (IBC) in which the proliferating epithelial cells remain within the boundaries of the ducto‐lobular system of the breast. DCIS is graded by pathologists using a three‐tier system: well differentiated (low nuclear grade, grade 1), intermediately differentiated (intermediate nuclear grade, grade 2), and poorly differentiated (high nuclear grade, grade 3). This histological assessment of grade is prognostic in terms of subsequent ipsilateral *in situ* and invasive lesion risk and is used to guide treatment decisions and to determine eligibility for inclusion in clinical trials. Although different guidelines are used to grade DCIS, there seems to be a substantial difference in interpretation (interobserver variability) in grading, even using the same guidelines [1]. Consequently, the prognostic and clinical value of DCIS grade is still a subject of debate [2, 3, 4]. There are, however, no other histological features or widely tested biomarkers presently available that can be used to predict reliably the progression of DCIS lesions to IBC [5]. Because of this uncertainty, almost all women with DCIS receive similar treatment to that given for IBC, i.e. mastectomy or breast‐conserving surgery (BCS) often supplemented by radiotherapy and/or endocrine therapy.

To investigate how to distinguish indolent from potentially hazardous DCIS and to be able to stratify DCIS based on risk of progression to invasive disease, we established the international PREvent ductal Carcinoma *In Situ* Invasive Overtreatment Now (PRECISION) initiative [6]. PRECISION synergises comprehensive prospective and retrospective DCIS studies [2, 4] and modelling and prospective clinical trials. Three ongoing prospective trials (COMET [7], LORIS [8], and LORD [9]) randomise patients between standard treatment and active surveillance for low‐risk DCIS. The identification of low‐risk DCIS based on morphological features is key not only for accrual into these trials but also for international collaborations for conducting research studies on DCIS. We embarked on a DCIS interobserver variability study using whole‐slide digital images of haematoxylin and eosin (H&E)‐stained sections of DCIS and including cohorts from three countries, namely, the USA, the UK, and the Netherlands (NL), that were reviewed by breast pathologists practicing in these three countries. Our primary goal was to evaluate the extent of interobserver variability in DCIS grading between pathologists from the same and from different health care systems. Subsequently, we aimed to assess possible causes for the variability and then address strategies to establish greater uniformity of grading.

## Methods

### Slide collection

Four institutions, The Netherlands Cancer Institute (NKI, NL), Kings College London (KCL, UK), MD Anderson Cancer Center (MDACC, USA), and Duke University Medical Center (DUMC, USA), participated in this study and contributed H&E‐stained whole‐slide images of tissue sections of DCIS. The cases were selected to represent the distribution of cytonuclear grade of DCIS (according to the pathology report or from previous review) in the participating countries or individual centres (see supplementary material, Table S1). The cases originated from the prospective, population‐based Sloane DCIS cohort (KCL, UK) [2]; the retrospective nation‐wide Dutch DCIS cohort [5]; and the retrospective, hospital‐based DUMC and MDACC cohorts. Whole‐slide images of one representative H&E‐stained section obtained from a formalin‐fixed paraffin‐embedded tissue block of a breast surgical resection were scanned at each centre, anonymised, and uploaded to the NKI and evaluated using the web‐based software platform Slidescore (see supplementary material, Table S1) [10]. To assess the number of slides that had to be evaluated, power calculations were performed (see supplementary material, Supplementary methods).

Local IRBs approved the use of the tissue blocks of NKI, MDACC, and Duke University with the waiver of informed consent because of the retrospective character of the study. For the UK slides held at Guy's and St Thomas' Hospitals in the King's Health Partner's Cancer Biobank facility, this is licensed by the Human Tissue Authority (license 12121). Ethics Committee approval was not required for this prospective cohort study originally conducted under the NHS Cancer Screening Program's application to the Patient Information Advisory Group.

### Histology and pathologists

To recapitulate pathology reporting in daily clinical practice, the breast pathologists interpreted the whole‐slide images of H&E tissue sections of DCIS without specific study‐related guidelines for all evaluated variables (see supplementary material, Table S2 for detailed information about the used diagnostic guidelines). The following histological variables were assessed (scoring form provided in supplementary material, Supplementary methods): presence of DCIS/atypical ductal hyperplasia/lobular carcinoma *in situ*, DCIS grade (1, 2, or 3), DCIS grade (low or high), dominant histological architecture (comedo/solid, cribriform, [micro]papillary, flat/clinging, and other), presence and semi‐quantitative frequency of mitosis (sparse and many), lymphocytic infiltrate (absent, subtle, and prominent), presence of calcifications (absent or present), presence of periductal fibrosis (absent, subtle, and prominent), and presence and type of necrosis (absent, present – comedo, present – focal, and present – comedo and focal).

Three breast pathologists from each country (NL, UK, and USA) evaluated all the slides independently. The participating pathologists completed a short questionnaire to collect information about their experience and criteria for DCIS grading that they followed in their clinical practice (see supplementary material, Table S3).

### Data analysis and statistics

The primary aim was the extent of variability between the nine pathologists for histological grade of DCIS based on review of the H&E‐stained slides. Tissue slides of insufficient quality, as judged by more than 50% of the participating pathologists for any histological variable, were excluded from analysis (*n* = 12).

As each slide was evaluated by each pathologist, generalised linear mixed models (GLMMs) for cross‐classified data structure were used to calculate kappa values as a chance‐corrected association between pathologists (*κ*_ma_) [11, 12]. *κ*_ma_ were obtained by taking into account levels of exact concordance, i.e. where pathologists assigned the exact same grade to a slide, and the level of disagreement among pathologists' classifications. *κ*_ma_ values were interpreted as the measurement of agreement using the criteria suggested by Landis and Koch [13], which are based on the interpretation that 0.00 is pure coincidence and 1.00 is perfect agreement: <0.00 as no, 0.00–0.20 as poor to slight, 0.21–0.40 as fair, 0.41–0.60 as moderate, 0.61–0.80 as substantial, and 0.81–1.00 as almost perfect agreement.

We modelled the histological variables separately, and to analyse the influence of the tissue slides' and pathologists' characteristics on each of the histological variables, GLMMs were adjusted for guidelines used, experience, country, and using the dominant or highest grade in case of heterogeneous DCIS as characteristics of the pathologists and origin of the slide (both country and centre) as characteristics of the slides. As all the pathologists from the same country used the same guidelines (except in the USA; see supplementary material, Table S3), including both ‘country of pathologists’ and ‘guidelines’ in the same multivariable model resulted in collinearity. We therefore chose to use the guidelines as a covariate instead of country to evaluate variation. The different values of *κ*_ma_ from the different adjusted models were compared to the results of the intercept‐only models. The ordinal package within the open‐source software R (version 2018; R Core Team, Vienna, Austria) was used for all the calculations.

### Majority opinion and influence of ER, PR, and HER2 expression

For each slide, the majority opinion classification, defined as the grade given by most of the pathologists, was assigned. When there was no majority opinion (i.e. equal number of pathologists, e.g. four pathologists graded 2, four pathologists graded 3, and one pathologist did not complete the form), the slide was assigned as not applicable (NA). The variable ‘number of pathologists’ was defined as the number of pathologists who make up the majority opinion and reflects the strength of agreement.

To investigate how to decrease interobserver variability, we retrospectively collected information about the status of oestrogen receptor (ER), progesterone receptor (PR), and overexpression of HER2 through immunohistochemical (IHC) stains obtained from whole slides from the NKI and the ER and PR status of the DUMC whole slides. MDACC had no IHC data available, and KCL assessed biomarker IHC on tissue microarrays, which was therefore excluded. For the IHC evaluated in NKI, ≥10% ER, ≥10% PR, and ≥10% strong membrane expression of HER2 were considered positive; for 2+ HER2 expression (equivocal), silver *in situ* hybridisation was performed. The IHC from USA (DUMC) was examined using the Allred method [14], and a score of >2 was considered positive (see supplementary material, Table S4 for more details about the scoring details, antibodies used, and IHC staining procedures).

## Results

### Cohort information and slide collection

Overall, 425 slides were provided by the participating centres (110 by NKI, KCL, and DUMC and 95 by MDACC). All slides were independently evaluated by the international group of nine breast pathologists. Of the 425 slides, 12 (2.8%) were excluded from all analyses based on quality issues as noted by the majority of the participating pathologists. For the histological variables of grade and mitoses, two and five additional cases, respectively, were excluded based on quality issues. The characteristics of both the included cases and the participating pathologists are given in supplementary material, Table S3.

### Differences between pathologists

**Figure 1:**
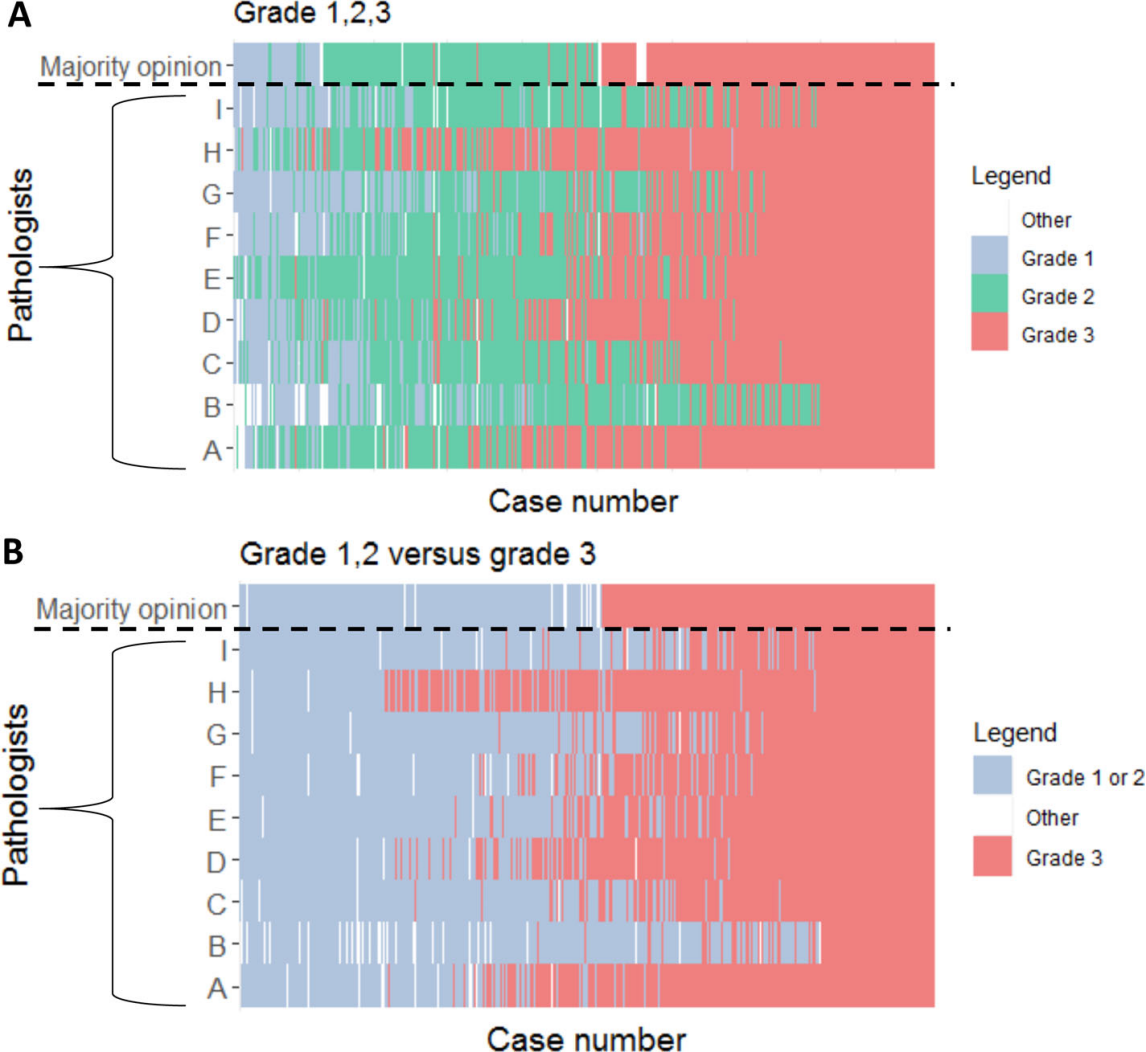
DCIS grades by pathologist (*y*‐axis) and by case (*x*‐axis). The upper row reflects the majority opinion. (A) Grade 1 or 2 or 3. (B) Grade 1 or 2 versus 3.

Figure 1A demonstrates both the individual evaluation and the majority opinion of grading as low (grade 1), intermediate (grade 2), and high (grade 3) per pathologist. It demonstrates substantial variability in grading the same lesion (see supplementary material, Figure S1 for histological examples of concordant and discordant slides). In addition, some pathologists had a tendency for lower grading, while others had a tendency for higher grading; variability diminished only slightly when grades 1 and 2 were grouped together (Figure 1B).

### Measure of associations between pathologists, *κ*_ma_

According to the GLMM, the probability that an individual H&E section of DCIS was classified as grade 1, 2, or 3 was 8%, 44%, and 48%, respectively. The model‐based chance‐corrected measure of association, *κ*_ma_, was estimated to be 0.50 (95% confidence interval [CI] 0.44–0.56; Table 1), indicating moderate association between the nine pathologists. For dichotomised grades 1 and 2 versus 3, the *κ*_ma_ also indicated moderate association (0.51; 95% CI 0.43–0.59). When the pathologists had to select between low or high grade as a binary grading system for all cases, the *κ*_ma_ was 0.52 (95% CI 0.45–0.59). The highest association was achieved for the category of dominant architectural pattern with a *κ*_ma_ of 0.61 (95% CI 0.57–0.64; Table 1), indicating substantial association.

**Table 1:** Model‐based measure of association (*κ*_ma_) for histological variables.

Histological variable	Model‐based weighted kappa (*κ*_ma_)	95% CI
DCIS grades 1, 2, and 3 (*n* = 411; intercept‐only model)	0.50	0.44–0.56
DCIS grades 1 and 2 versus 3 (*n* = 411)	0.51	0.43–0.59
DCIS grade 1 versus 2 and 3 (*n* = 411)	0.45	0.41–0.50
DCIS grade as binary, low versus high (*n* = 411)	0.52	0.45–0.59
Necrosis; absent versus present (*n* = 413, manually dichotomised)	0.55	0.51–0.59
Calcifications; absent versus present (*n* = 413)	0.51	0.48–0.55
Lymphocytic infiltrate; absent versus subtle versus prominent (*n* = 413)	0.47	0.38–0.55
Periductal fibrosis; absent versus subtle versus prominent (*n* = 413)	0.35	0.03–0.31
Mitoses; sparse versus many (*n* = 408)	0.33	0.24–0.42
Architectural pattern; solid and comedo versus cribriform, flat, and (micro)papillary (*n* = 413)	0.61	0.58–0.64

DCIS grade 1 denotes low grade, 2 intermediate grade, and 3 high grade.

When incorporating guidelines used as a covariate at the pathologist level, the *κ*_ma_ in the univariable GLMM for DCIS grade did not change in comparison to the intercept‐only model (*κ*_ma_ = 0.53; 95% CI 0.48–0.57; *p* = 0.52; Table 2). We aimed to investigate whether the *κ*_ma_ improved when we only included pathologists using the same guideline in the GLMM. A minimum of three observers was necessary, enabling us to analyse the UK and World Health Organization (WHO) guidelines. Pathologists utilising the UK pathology guideline had better association between each other (*κ*_ma_ = 0.58; 95% CI 0.56–0.61) compared to pathologists using the WHO guidance, which showed a *κ*_ma_ of 0.48 (95% CI 0.36–0.61; *p* = 0.80), and a model including the use of the UK pathology guideline shows better association between pathologists compared to the standard model (*p* = 0.02).

**Table 2:** Univariable and multivariable analyses with pathological and histological features as covariates to determine the influence on DCIS grade (1 or 2 or 3).

Variable	Model‐based weighted kappa (*κ*_ma_)	95% CI	*P* value for kappa comparison with the outcome only
**DCIS grade 1, 2, or 3**	0.50	0.44–0.56	
**Univariable analysis – adjusted for features of the pathologists**			
Experience	0.50	0.44–0.57	0.95
Country of pathologist	0.51	0.44–0.57	0.91
Heterogeneous DCIS; highest versus most prominent versus other	0.53	0.48–0.57	0.54
Guideline used	0.53	0.48–0.57	0.52
Split according to guideline used			
1 Consensus Conference	Only used by one pathologist, not possible		
2 UK Royal College of Pathologists	0.58	0.56–0.61	0.02*
3 College of American Pathologists	Only used by two pathologists, not possible		
4 WHO	0.48	0.36–0.61	0.80
**Univariable analysis – histological features**			
Necrosis; absent versus present	0.45	0.39–0.52	0.31
Calcification; absent versus present	0.50	0.44–0.57	0.97
Lymphocytic infiltrate; absent versus subtle versus prominent	0.46	0.41–0.52	0.37
Periductal fibrosis; absent versus subtle versus prominent	0.48	0.43–0.54	0.72
Mitoses; sparse versus many	0.46	0.40–0.52	0.40
Architectural pattern; solid and comedo versus. cribriform, flat, and (micro)papillary	0.45	0.39–0.52	0.33
**Multivariable analysis – adjusted for features of the pathologists**			
Guidelines + experience + solution to heterogeneity of DCIS	0.57	0.54–0.59	0.06
Country + experience + solution to heterogeneity DCIS	0.53	0.49–0.58	0.41
**Multivariable analysis –adjusted for histological features**			
Necrosis + calcification + lymphoid infiltrate + periductal fibrosis + mitosis + architectural pattern	0.31	0.26–0.36	<0.01*

DCIS grade 1 denotes low grade, 2 intermediate grade, and 3 high grade.

^*^ *P* value showing a significant effect, i.e. *p* < 0.05.

For DCIS cytonuclear grading, the associations between pathologists did not change when the following covariates were separately added to the model on the pathologist and case levels: pathologist's experience (*κ*_ma_ = 0.50; 95% CI 0.44–0.57), country of the pathologist (*κ*_ma_ = 0.51; 95% CI 0.44–0.57), and country of origin of the case (*κ*_ma_ = 0.49; 95% CI 0.42–0.55). When the model was adjusted for additional histological variables separately, the *κ*_ma_ for DCIS nuclear grade did not improve (Table 2). Multivariable modelling including the variables characterising the pathologists (i.e. use of guidelines, experience, and manner of reporting cases of heterogeneous DCIS) showed an increased but not statistically improved *κ*_ma_ of 0.57 (95% CI 0.55–0.60; *p* = 0.06). When the model was adjusted for all other histological variables together, the reproducibility for DCIS grading decreased (*κ*_ma_ = 0.31; 95% CI 0.26–0.36; Table 2).

### Majority opinion and influence of ER and HER2 expression

**Figure 2:**
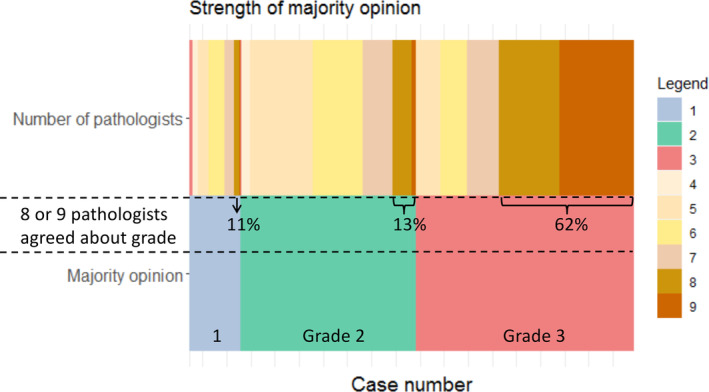
The strength of the majority opinion for low, intermediate, and high grade. The bottom row shows the distribution of DCIS grade according to the majority opinion and the upper row the number of pathologists that represent the majority opinion.

**Figure 3:**
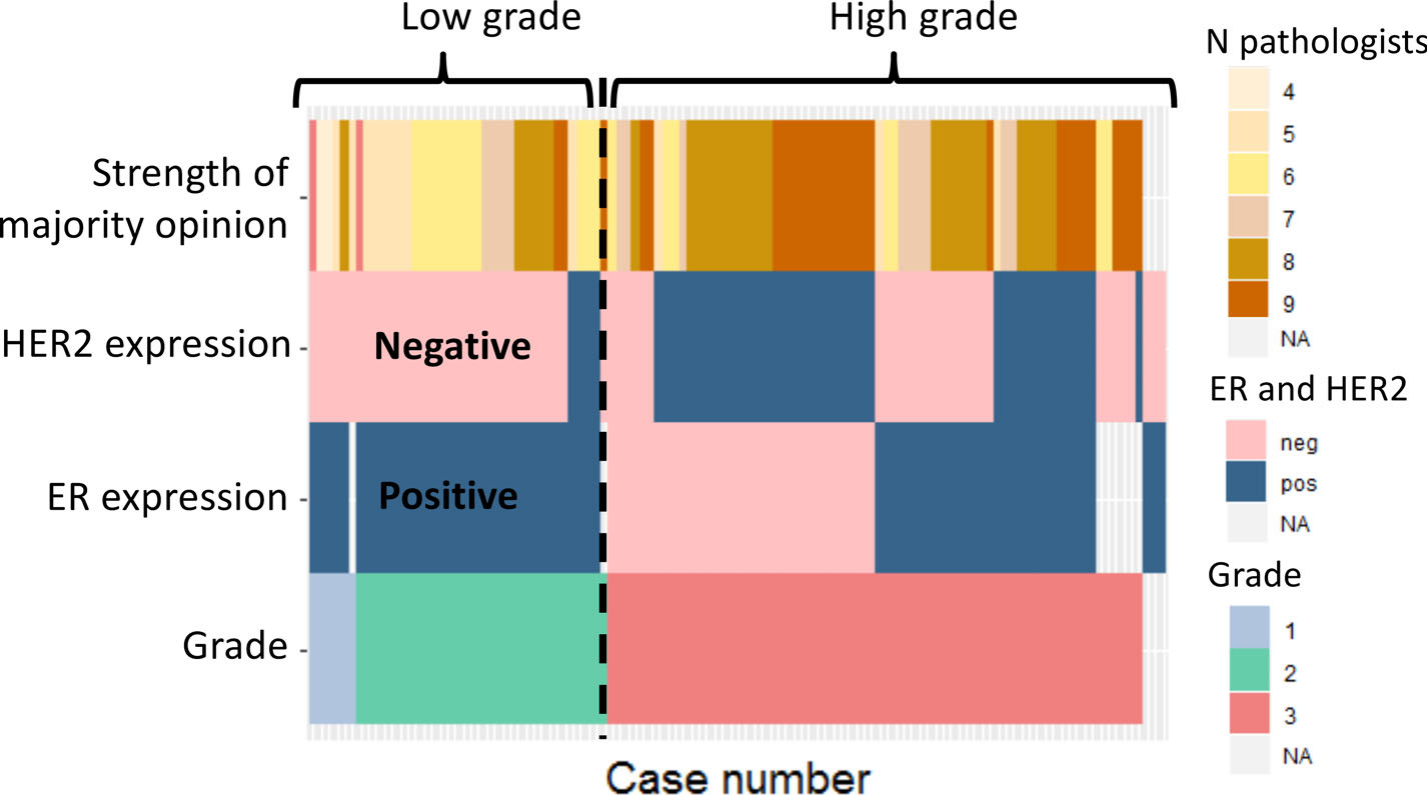
ER and HER2 expression in relation to low (grade 1), intermediate (grade 2), and high (grade 3) grade according to the majority opinion and the strength of the majority opinion including the NL (NKI) cases (*n* = 110) only. neg, negative; pos, positive.

Grade 3 DCIS showed less variability than grade 1 or grade 2 disease: 62% of lesions were scored by eight or nine pathologists as grade 3 (Figure 2). We then explored whether ER and/or HER2 expression could help in the identification of grade 3 (high‐grade) lesions (see Figure 3 and supplementary material, Table S5). Figure 3, representing only NKI cases (*n* = 106), shows that lesions categorised as grade 1 DCIS by the majority opinion were all ER positive and HER2 negative, and those categorised as grade 2 were predominantly ER positive (100%) and HER2 negative (88%). Grade 3 DCIS cases, determined by the majority opinion, were heterogeneous for ER and HER2 expression, with both positive and negative cases represented. We were able to validate the results of ER expression in the IHC data from DUMC (USA) (see supplementary material, Table S5); none of the low‐grade cases of DCIS according to majority opinion were ER negative.

## Discussion

Although reproducibility of the *diagnosis* of DCIS has been demonstrated to have substantial agreement [15], this international study among nine pathologists showed kappa values of 0.5–0.6 for the assessment of DCIS *grade* based on a GLMM, indicating only a moderate association between pathologists. Including guidelines as a covariate in the GLMM did not improve the association; analysing the data specifically for the UK pathology guidelines [16] showed a statistically significant improvement in associations between pathologists compared to the standard model. Linking the interobserver variability data to IHC stains demonstrated that almost all non‐high‐grade DCIS lesions according to the majority opinion were ER positive (100%) and HER2 negative (89%), whereas 55% of high‐grade DCIS lesions were ER negative and/or HER2 positive (62%). Applying these biomarker stains might be helpful to prevent accidental selection of high‐grade DCIS, e.g. in active surveillance protocols.

The significance of cytonuclear grade of DCIS, while generally regarded as a predictor of risk of recurrence as subsequent *in situ* or invasive disease [2, 17], is not universally accepted [3, 4]. Here, we show variability in grading DCIS; 20% of cases were highly discordant as different pathologists categorised the exact same lesion on a single identical H&E scanned slide as grade 1, 2, or 3. This discrepancy might result in a low correlation between prognosis and grade. Multiple studies have shown high inter‐rater variability of DCIS grade and have suggested methods for improvements in consistency, such as dichotomous scoring [18, 19, 20], assessing the proportions of DCIS heterogeneity [21], adding uniform e‐learning [22], and using second opinions [23]. Our results are based on a GLMM taking into account that the same pathologists examined the same slides [24]. Such variability in grading of DCIS has profound consequences for the inclusion of cases of DCIS in active surveillance trials (COMET [7], LORIS [8], and LORD [9]), where low or intermediate grade (or low and lower portions of intermediate grade in LORIS) is the inclusion criterion. Regarding COMET and LORD, where no central review is performed, patients are deemed eligible or ineligible based on examination by an individual local pathologist. For all these reasons, it is essential to achieve a globally reproducible scoring system.

As noted, some pathologists tended to score substantially more DCIS lesions as low grade than others, while the opposite also occurred. In the case of heterogeneous DCIS, one pathologist categorised the lesion according to the most prominent grade, while the majority (7/9) classified the DCIS by the highest cytonuclear grade present, which could explain some of the differences observed. One guideline (UK) clarifies that the highest grade should be recorded when, uncommonly, more than one form is present [16]. Other previous guidelines such as the 2012 WHO [25] or 1997 Consensus conference [26] have advised that all grades present should be noted. In this study, we specifically sought to simulate daily clinical practice and therefore did not provide specific guidelines beforehand for grading or for any of the other histological features recorded. Compared to the standard model, pathologists who followed the UK pathology guidelines [16] showed significantly more mutual concordance (*κ*_ma_ = 0.58; *p* = 0.02; Table 2) than those who used the 2012 WHO guidance [25] (κ_ma_ = 0.48; *p* = 0.80). However, when exploring the details of the various guidelines, no major differences were apparent that could explain the better concordance for the UK guideline compared to the others [25, 26, 27] (see supplementary material, Table S2). In the UK, adherence to the breast reporting guidelines is mandated for breast screening pathologists, as is participation in a twice‐yearly national breast external quality assurance slide review scheme (that includes cases of DCIS), as well as attendance at regional meetings to discuss these. However, two of the three UK breast pathologists are central reviewers in the LORIS trial (through which they have also provided advice and educational webinars for other UK pathologists) and two work in the same department (albeit where cases are reported by the individual). It is therefore difficult to know if the greater concordance of the three UK pathologists represents the recent focus on consistency of grading of DCIS in the UK; the overall educational and quality assurance mechanisms in place; or simply that they have had the opportunity to work together, discuss problematic cases, and align their approach to DCIS grading. Nevertheless, this supports the use of one international DCIS grading system along with a uniform training programme, as also suggested by other studies [1, 18, 19, 20, 28].

To improve guidance for clinical decision‐making, we explored the use of IHC. In our data on the NKI series, majority‐opinion low‐ and intermediate‐grade DCIS was characterised by ER positivity and HER2 negativity. We were able to validate this in DUMC (USA) slides for ER expression, scored by an alternative (Allred [14]) method (see supplementary material, Table S5). This is in line with other studies which also showed that ER was frequently expressed in low‐ and intermediate‐grade DCIS, whereas HER2 positivity was much more frequent in high‐grade disease [29, 30]. The proportion of pure DCIS that is ER positive is 68–83% [5, 29, 30, 31, 32], while HER2 positivity ranges from 25 to 35% [5, 30, 31, 33]. IHC scoring for ER and HER2 is reported to have high interobserver agreement between pathologists (intraclass coefficient > 0.8) [5], which is better than the interobserver agreement for grade (presented here and in other studies [18, 19, 20, 21, 34, 35, 36]). Globally, the use of IHC within DCIS is variable; no marker is currently included in the international DCIS pathology minimum data sets, although in some national data sets (e.g. USA), ER assessment is mandated. In the USA, half of the patients with ER‐positive DCIS are treated with endocrine therapy [37], but this is still a subject of debate, and this value is much lower in other countries [2, 3, 4]. Positive ER/PR and negative HER2 status is used in the COMET trial as inclusion criteria for the active surveillance regimen [7] in keeping with the data presented here; when DCIS shows ER negativity and/or HER2 positivity, classification as high‐grade DCIS should be considered.

The present study has several limitations. First, only limited outcome data were available for many of the cases, and therefore, the primary outcome was histological interobserver variability instead of recurrence or progression of disease. Unfortunately, we were unable to validate the results of the 106 NKI cases in another cohort. To our knowledge, only one single‐centre study has correlated interobserver variability with progression to IBC and found that using majority opinion‐based scores of grade (grade 1 + 2 versus 3), mitotic activity, and growth pattern was associated with outcome in patients treated with BCS only and not in patients treated with BCS plus radiotherapy. Furthermore, we sought to simulate daily clinical practice and therefore did not require adherence to guidelines assigned specifically for the study. The concordance may have been better if we had provided guidance for assessment of the slides. It should also be noted that most of the study pathologists do not use digital slides to diagnose cases in their daily practice, although digital pathology will become daily practice in the near future. In this study, a DCIS case was represented by one slide, while in daily practice, multiple slides are typically examined in evaluating DCIS. Moreover, increasing the number of (international) pathologists would have provided more information about the differences between countries and the guidelines used. Finally, independent validation of the data on ER and HER2 expression presented is necessary in order to prove the association between low‐ and intermediate‐grade DCIS and IHC ER positivity and HER2 negativity.

The strength of this study is the international character of both the cases of DCIS and the participating pathologists. Moreover, the data have been analysed using a method that takes into account the cross‐classified data structure.

In conclusion, in this international study, we show a moderate concordance for a range of histological features of DCIS between nine specialist breast pathologists. As cytonuclear grade of DCIS plays a role as a prognostic parameter in treatment decisions, there is an urgent need for the adherence of pathologists to a more objective scoring system. As a first step in improving reproducibility, we suggest that ER negativity and/or HER2 positivity of an individual DCIS lesion is indicative of a high‐grade lesion, which may be of value in distinguishing this from low‐ and intermediate‐grade DCIS, although validation is required.

## Authors contributions statement

MvS, SEP, SK, AH, HS, AT, EHL and JW conceived and designed the study. JH provided IT support. KJ and MS provided statistical support. MvS, SEP, IB, SK, AH, AT, JSJT, WN, LCC, DC, JonB, JooB, JW and EHL collected and assembled data. MvS, KJ, SEP, AH, SK, AT, HS, MS, EHL and JW analysed and interpreted data. All authors wrote and had final approval of the manuscript.

## Supporting information


**Supplementary methods**
**Figure S1.** Histological examples of concordant and discordant slides
**Table S1.** Information regarding included slides
**Table S2.** Histological criteria of the guidelines used
**Table S3.** Characteristics of participating pathologists
**Table S4.** Characteristics of antibodies used
**Table S5.** ER, PR, and HER2 expression in relation to interobserver variability

## Data Availability

The data generated and analysed during this study will be available from the corresponding author upon reasonable request.
