# Supplementary material for: Variability in grading of ductal carcinoma in situ among an international group of pathologists
Source: J Pathol Clin Res. 2021 Feb 23;7(3):233–42. doi: 10.1002/cjp2.201 (PMC8073001; doi:10.1002/cjp2.201)
Supplement: Supplementary file 1 — Supplementary methods Figure S1. Histological examples of concordant and discordant slides Table S1. Information regarding included slides Table S2. Histological criteria of the guidelines used Table S3. Characteristics of participating pathologists Table S4. Characteristics of antibodies used Table S5. ER, PR, and HER2 expression in relation to interobserver variability [file CJP2-7-233-s001.docx]

**Variability in grading of ductal carcinoma in situ among an international group of pathologists**

M van Seijen *et al*. *J Pathol Clin Res* DOI: 10.1002/cjp2.201

**Supplementary Material**

**The reference number refers to the list in the main paper**

**Supplementary Methods**

**Figure S1.** Histological examples of concordant and discordant slides

**Table S1.** Information regarding included slides

**Table S2.** Histological criteria of the guidelines used

**Table S3.** Characteristics of participating pathologists

**Table S4.** Characteristics of antibodies used

**Table S5.** ER, PR and HER2 expression in relation to interobserver variability

**Supplementary Methods**

***Power Calculation method***

Based on a statistical power calculation, we aimed to have at least 379 tissue slides all evaluated by nine pathologists, i.e. three pathologists from each country. This number was obtained taking into consideration the proportion of high-grade DCIS anticipated between three countries and taking correlations within pathologists from the same country into account. We expected overall proportions of high-grade DCIS from the NL, US and UK to be 42%, 52% and 62% respectively, and we assumed that correlations between randomly chosen grades of DCIS within pathologists from the same country was 0.60. Such a design would give us at least 80% power to detect all pairwise comparisons of proportions between different countries using a corrected for multiple testing significance level of 0.016.

***Scoring form for evaluating the DCIS slides***

- **Disease present: DCIS**
- **Disease present: ADH**
- **Disease present: LCIS**

**Dominant architectural pattern:**

- Not assessable
- Comedo
- Solid
- Cribriform
- Flat/Clinging
- (Micro)papillary

**Calcification present:**

- Not assessable
- Absent
- Present

**Necrosis present:**

- Not assessable
- Absent
- Present: Comedo
- Present: Focal
- Present: Comedo and focal

**Periductal fibrosis present:**

- Not assessable
- Absent
- Subtle
- Prominent

**Lymphocytic infiltrate (in relation with DCIS) present:**

- Not assessable
- Absent
- Subtle
- Prominent

**Histological grade DCIS (1/2/3):**

- Not assessable
- Low grade
- Intermediate grade
- High grade

**Histological grade DCIS (low/high):**

- Not assessable
- Low grade
- High grade

**Frequency of mitoses:**

- Not assessable
- Sparse
- Many

**Comments (other diagnosis or otherwise):** *open text field*

***
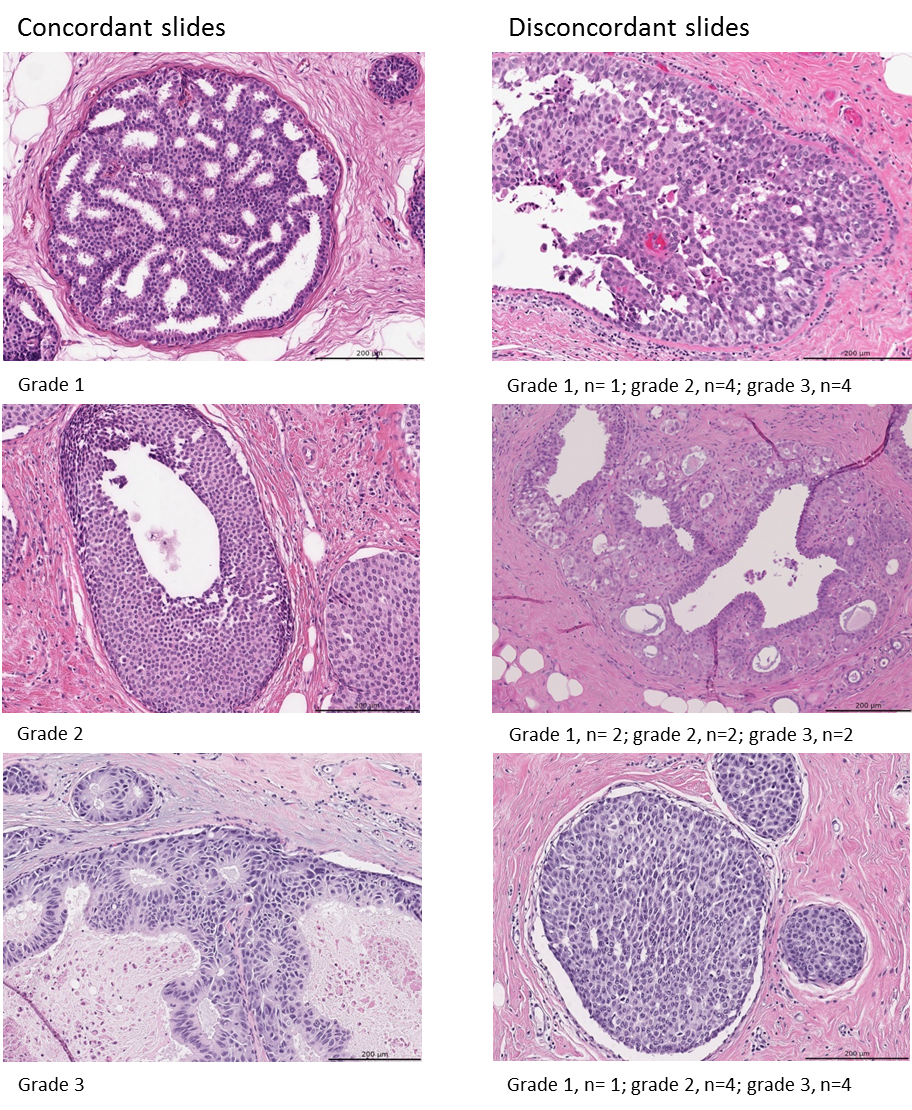
***

**Figure S1.** Histological examples of concordant and discordant slides.

**Table S1.** Information regarding included slides.

Before inclusion in the study, all slides were evaluated to ensure that they were in focus. A pilot study was performed to check if the quality between the slides from the different centres was similar.

|  | **UK (KCL)** | **NL (NKI)** | **USA (Duke)** | **USA (MDACC)** |
| --- | --- | --- | --- | --- |
| **Type of scanner** | NanoZoomer 2.0 HT Slide Scanner (Hamatsu Photonics) | Aperio AT2 Slide Scanner (Leica Biosystems) | Leica Aperio scanner | Aperio AT2 Slide Scanner (Leica Bio systems) |
| **Magnification** | 40x | 20x | 20x | 20x |
| **Type of slides** | Whole breast images | Whole breast images | Whole breast images | Whole breast images |
| **Format** | .ndpi | .svs | .svs | .svs |
| **Grade to original pathology reports**  **(N, %)** |  |  |  |  |
| Grade 1 | 12 (11%) | 19 (17%) | 5 (5%) | 14 (16%) |
| Grade 2 | 34 (31%) | 36 (33%) | 41 (45%) | 38 (45%) |
| Grade 3 | 64 (58%) | 55 (50%) | 46 (50%) | 33 (39%) |
| Excluded* | 0 | 0 | 18 | 10 |

* Slides originally evaluated as grade 1-2 or 2-3 were excluded

**Table S2**. Histological criteria of the guidelines used.

|  | **WHO 2012** | **RCPath Guidelines UK** | **College of American pathologists (Lester et al)** | **Consensus conference on classification of DCIS** |
| --- | --- | --- | --- | --- |
| **Grade 1 (low)** |  |  |  |  |
| Cell appearance | Small monomorphic cells with regular chromatin inconspicuous nucleoli | Monomorphic, evenly spaced cells with rounded, centrally placed nuclei and inconspicuous nucleoli | Monomorphic cells, usually diffuse finely dispersed chromatin, only occasional nucleoli | Monomorphic, usually exhibit diffuse, finely dispersed chromatin, only occasional nucleoli and mitotic figures |
| Pattern | Arcades, micropapillae, cribriform, or solid | Generally arranged in micropapillary and cribriform patterns |  |  |
| Orientation | Polarised cells around rosettes | Usually polarisation of cells covering the micropapillae | Polarised towards luminal spaces | Usually associated with polarisation of constituent cells |
| Nuclear Size | Nuclear: Uniform size, | Nuclear: 2x-3x erythrocyte | Nuclear: 1x-2x size of normal RBC or normal duct epithelial cell nucleus | Nuclear: 1.5-2.0 RBC or duct epithelial cell nuclear dimensions |
| Mitosis | Rare | Few |  |  |
| Necrosis | uncommon | rarely individual cell necrosis |  |  |
| Calcifications | Often psammomatous type |  |  |  |
| **Grade 2 (intermediate)** |  |  |  |  |
| Cell appearance | Mild to moderate variability in shape, variably coarse chromatin, variably prominent nucleoli | Moderate pleomorphism, nuclear to cytoplasmic ratio is often high, and one or two nucleoli may be identified. one or two nucleoli. Clear cell or apocrine types often fall into this category | Intermediate pleomorphism, intermediate chromatin, nucleoli | Nuclei that are neither NG1 or NG2 |
| Pattern |  | Solid, cribriform or micropapillary. |  |  |
| Orientation | Cell polarisation is not well developed as in low-nuclear grade | some degree of polarisation | Intermediate polarisation |  |
| Size | Nuclear: variability in size | Nuclear: 2-3x size of an erythrocyte | Nuclear: intermediate |  |
| Mitosis | Maybe present |  | Intermediate |  |
| Calcifications | Distribution of amorphous of or laminated microcalcifications is generally similar to low-nuclear-grade |  |  |  |
| Necrosis | Punctate or comedo necrosis maybe present |  |  |  |
| **Grade 3 (high)** |  |  |  |  |
| Cell appearance | Highly atypical cells with pleiomorphic nuclei | pleomorphic, irregularly spaced and, nuclei exhibiting marked variation in size with irregular nuclear contours, coarse chromatin and prominent nucleoli | Markedly pleomorphic, usually vesicular with irregular chromatin distribution, prominent nucleoli | Markedly pleiomorphic, usually vesicular and exhibit irregular chromatin distribution and prominent often multiple nucleoli |
| Pattern | Solid, cribriform or micropapillary patterns | It is often solid with comedo-type central necrosis. Also micropapillary and cribriform patterns frequently associated with central comedo type necrosis |  |  |
| Orientation | Poorly polarised | rarely any polarisation of cells | Usually not polarised towards the luminal space |  |
| Size | Lesion: usually >5mm | Nuclear: >3x the size of erythrocytes | Nuclear : >2.5x size of RBC or normal duct epithelial cell nucleus | Nuclear: usually>2.5 x RBC or duct epithelial cell nuclear dimensions |
| Mitosis | Usually common (not required) | usually frequent and abnormal forms may be seen |  | Might be conspicuous |
| Calcifications | Amorphous microcalcifications are common and usually associated with intraluminal debris |  |  |  |
| Necrosis | Frequently presence of comedo necrosis (not obligatory) |  |  |  |

**Table S3.** Characteristics of participating pathologists and examined tissue slides.

| ***Pathologists*** | **Number (%)** | ***Slides*** | **Number (%)** |
| --- | --- | --- | --- |
| **Total** | **9 (100%)** | **Total** | **425 (100%)** |
| Country |  | Centre |  |
| The Netherlands (NL) | 3 (33%) | NKI | 110 (26%) |
| United Kingdom (UK) | 3 (33%) | KCL | 110 (26%) |
| United States (US) | 3 (33%) | Duke | 110 (26%) |
|  |  | MDACC | 95 (22%) |
| Experience |  | Grade according to majority opinion | 399 (100%) |
| Median | 12.0 years | 1 | 45 (11%) |
| <10 yrs | 5 (56%) | 2 | 158 (40%) |
| >=10 yrs | 4 (44%) | 3 | 196 (49%) |
| Guidelines |  |  |  |
| WHO | 3 (33%) |  |  |
| UK RCPath Guidelines | 3 (33%) |  |  |
| College of American pathologists | 2 (22%) |  |  |
| Consensus conference on classification of DCIS | 1 (11%) |  |  |
| In case of heterogeneous DCIS | |  |  |
| Highest grade | 7 (78%) |  |  |
| Most prominent grade | 1 (11%) |  |  |
| Other | 1 (11%) |  |  |

**Table S4.** Details of the scoring of the immunohistochemical stains and characteristics of the antibodies used.

|  | **Antigen** | **NL (NKI)** | **USA (Duke)** |
| --- | --- | --- | --- |
| **Clone** | ER | SP1 | 1D5 and ER-2-123 |
|  | PR | 1E2 | PgR1294 |
|  | HER2 | 4B5 | *Not used in this study* |
| **Dilution** | ER | ready-to-use | ready-to use |
|  | PR | ready-to-use | ready-to-use |
|  | HER2 | ready-to-use | *Not used in this study* |
| **Manufacturer** | ER | Ventana medical systems | DAKO / Agilent |
|  | PR | Ventana medical systems | Dako / Agilent |
|  | HER2 | Ventana medical systems | *Not used in this study* |
| **Type of slides** |  | Whole slides | Whole slides |
| **Scorings method** | ER | % of positive cells; ≥10% is positive | Allred method; >2 is considered as positive |
|  | PR | % of positive cells; ≥10% is positive | Allred method; >2 is considered as positive |
|  | HER2 | % membrane staining; ≥10% is positive(3+), if incomplete or weak (2+) SISH was performed | *Not used in this study* |
| **Number of observers** |  | 7 (5 pathologists) | 1 out of 5 breast pathologists |
| **More details** |  | Supplementary table, Visser et al. [38] |  |

**Table S5.** ER,PR and HER2 expression in relation to interobserver variability in a subset. Grade 1, grade 2, or grade 3 are established according to the majority opinion. For 'certain' cases eight or nine pathologists agreed and for 'uncertain' cases <8 pathologists agreed.

| **NL** | **ERneg** | **ERpos** |  | **PRneg** | **PRpos** |  | **HER2neg** | **HER2pos** |  |
| --- | --- | --- | --- | --- | --- | --- | --- | --- | --- |
| **Grade 1** | **0** | **5** | **5** | **0** | **5** | **5** | **6** | **0** | **6** |
| *Certain (8,9)* | 0 | 1 |  | 0 | 1 |  | 1 | 0 |  |
| *Uncertain (<8)* | 0 | 4 |  | 11 | 4 |  | 5 | 0 |  |
| **Grade 2** | **0** | **31** | **31** | **13** | **18** | **31** | **28** | **4** | **32** |
| *Certain (8,9)* | 0 | 7 |  | 2 | 5 |  | 8 | 0 |  |
| *Uncertain (<8)* | 0 | 24 |  | 11 | 13 |  | 20 | 4 |  |
| **Grade 3** | **34** | **28** | **62** | **47** | **15** | **62** | **26** | **42** | **68** |
| *Certain (8,9)* | 27 | 18 |  | 34 | 11 |  | 14 | 35 |  |
| *Uncertain (<8)* | 7 | 10 |  | 13 | 4 |  | 12 | 7 |  |
| **Total** | **34** | **64** | **98** | **60** | **38** | **98** | **60** | **46** | **106** |
| **USA (Duke)** | **ERneg** | **ERpos** |  | **PRneg** | **PRpos** |  |  |  |  |
| **Grade 1** | **0** | **8** | **8** | **0** | **8** | **8** |  |  |  |
| *Certain (8,9)* | 0 | 0 |  | 0 | 0 |  |  |  |  |
| *Uncertain (<8)* | 0 | 8 |  | 0 | 8 |  |  |  |  |
| **Grade 2** | **0** | **46** | **46** | **2** | **42** | **44** |  |  |  |
| *Certain (8,9)* | 0 | 5 |  | 0 | 5 |  |  |  |  |
| *Uncertain (<8)* | 0 | 41 |  | 2 | 37 |  |  |  |  |
| **Grade 3** | **16** | **33** | **49** | **21** | **26** | **47** |  |  |  |
| *Certain (8,9)* | 14 | 15 |  | 17 | 12 |  |  |  |  |
| *Uncertain (<8)* | 2 | 18 |  | 4 | 14 |  |  |  |  |
| **Total** | **16** | **87** | **103** | **23** | **76** | **99** |  |  |  |
